# Supplementary figures and images for: Multi-tissue epigenetic analysis of the osteoarthritis susceptibility locus mapping to the plectin gene PLEC
Source: Osteoarthritis Cartilage. 2020 Nov;28(11):1448–58. doi: 10.1016/j.joca.2020.06.001 (PMC7594932; doi:10.1016/j.joca.2020.06.001)

Supplementary Figure 1

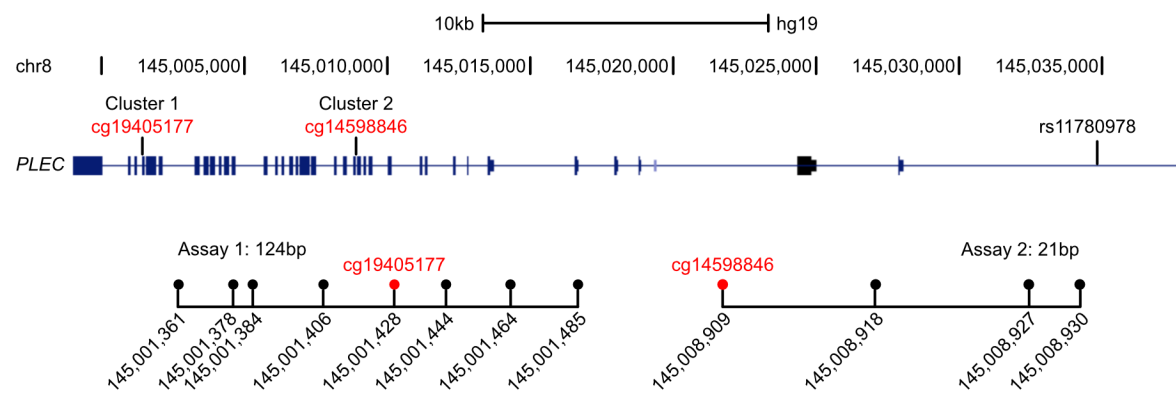

Supplement: Supplementary Fig. 1 — The location in PLEC of rs11780978 and the analysed CpGs. The PLEC gene is shown in blue, exons are boxes/vertical lines, and the direction of transcription is from right to left. The cg19405177 assay captures a total of 8 CpGs, the cg14598846 assay a total of four. cg19405177 and cg14598846 are marked as red circles, the additional CpGs as black circles. The physical position of the CpGs on chromosome 8 (hg19 assembly, UCSC Genome Browser) is shown. [file mmc3.pdf]

Supplementary Figure 2

A

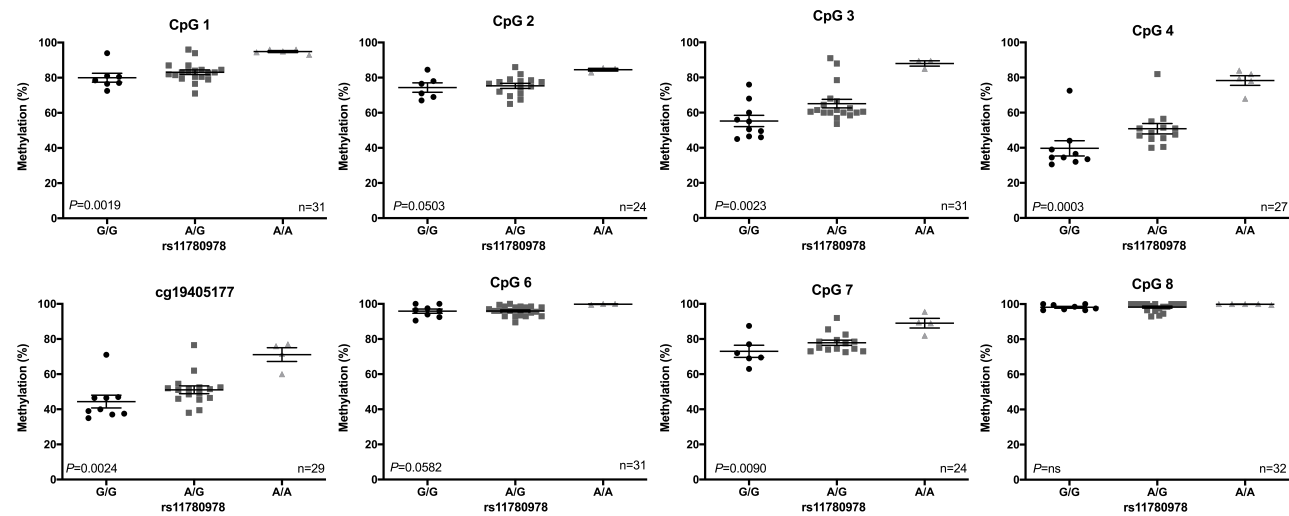

B

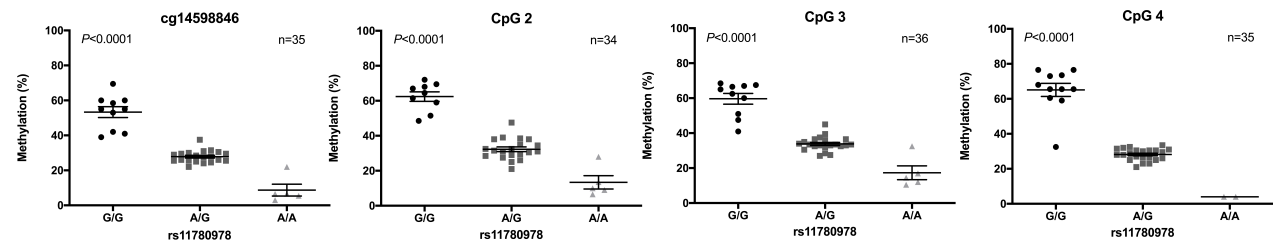

Supplement: Supplementary Fig. 2 — Association between rs11780978 genotype and methylation at CpG clusters cg19405177 (A) and cg14598846 (B) in cartilage DNA. P values were calculated using the Kruskal–Wallis test. Horizontal lines and error bars show the mean ± SEM. n = the number of patients providing data per CpG site. [file mmc4.pdf]

Supplementary Figure 3

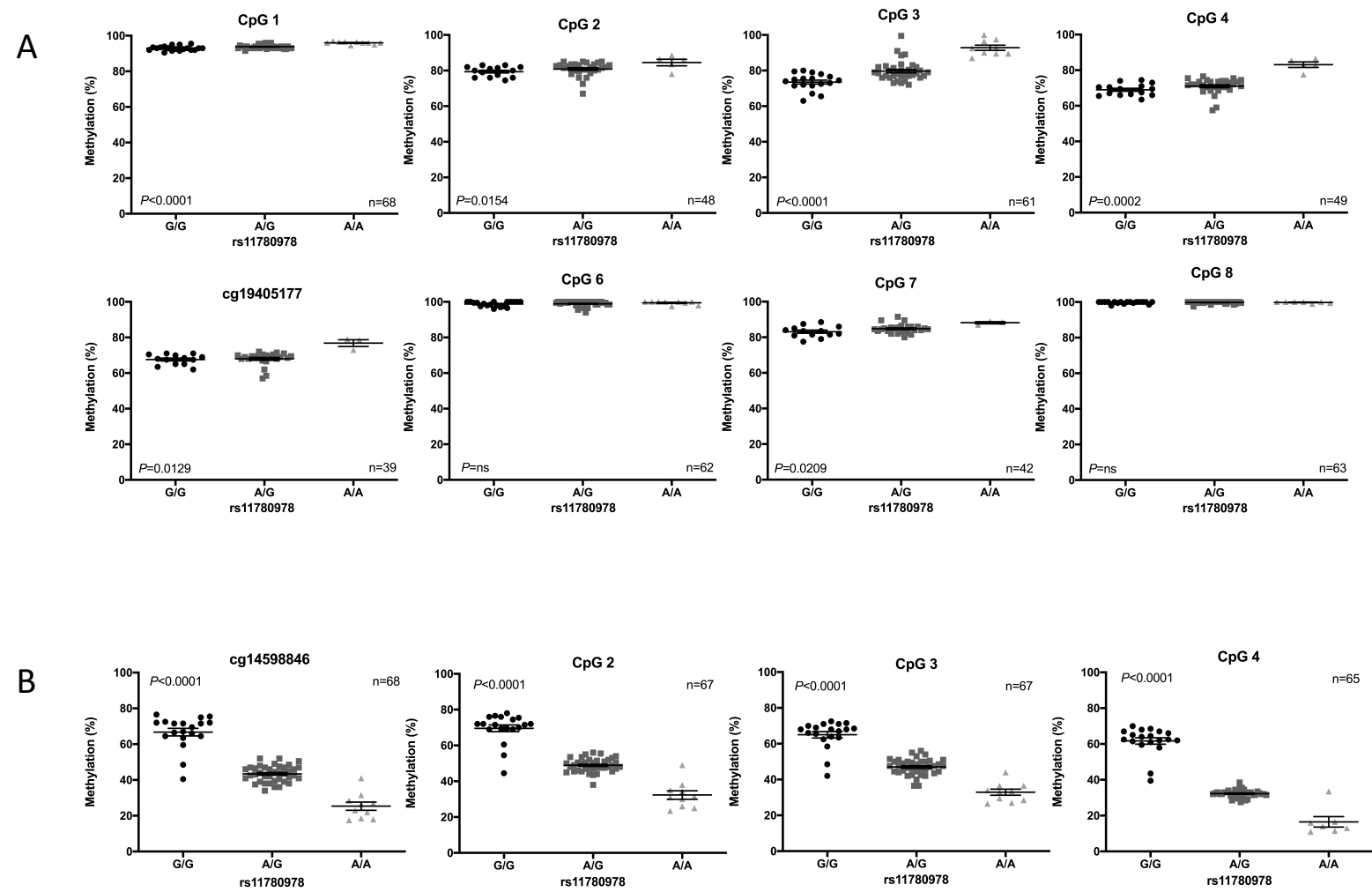

Supplement: Supplementary Fig. 3 — Association between rs11780978 genotype and methylation at CpG clusters cg19405177 (A) and cg14598846 (B) in fat pad DNA. P values were calculated using the Kruskal–Wallis test. Horizontal lines and error bars show the mean ± SEM. n = the number of patients providing data per CpG site. [file mmc5.pdf]

Supplementary Figure 4

A

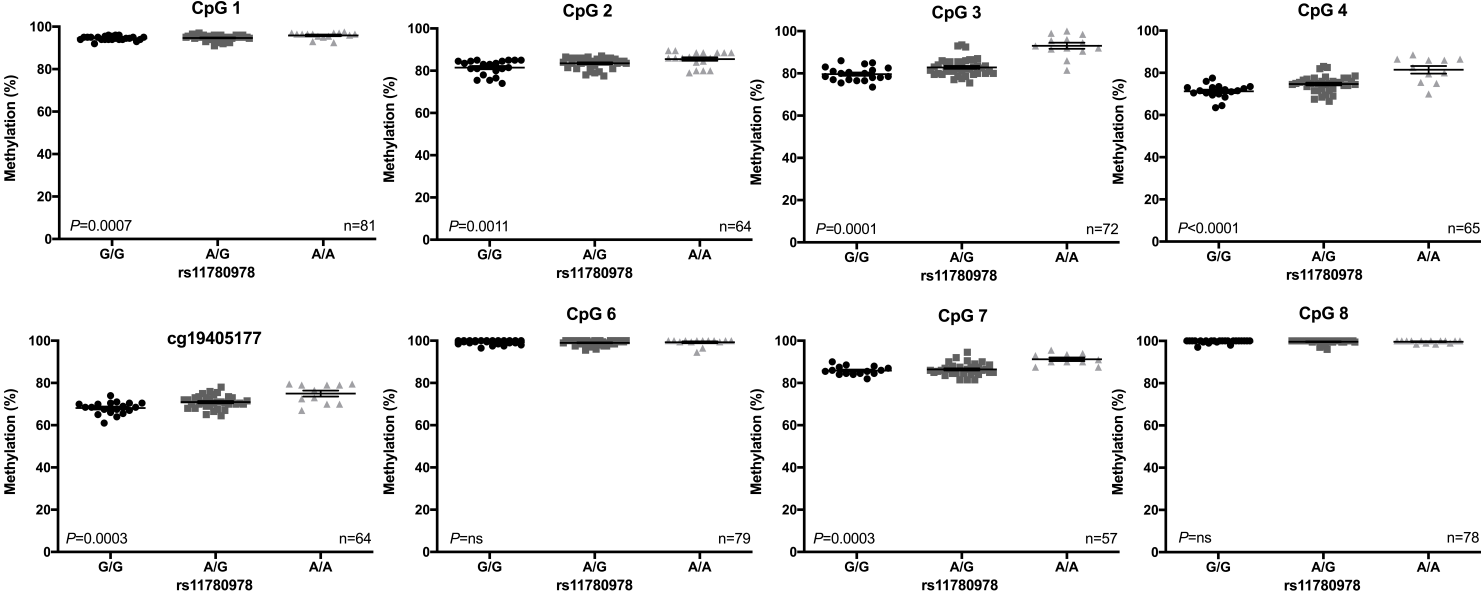

B

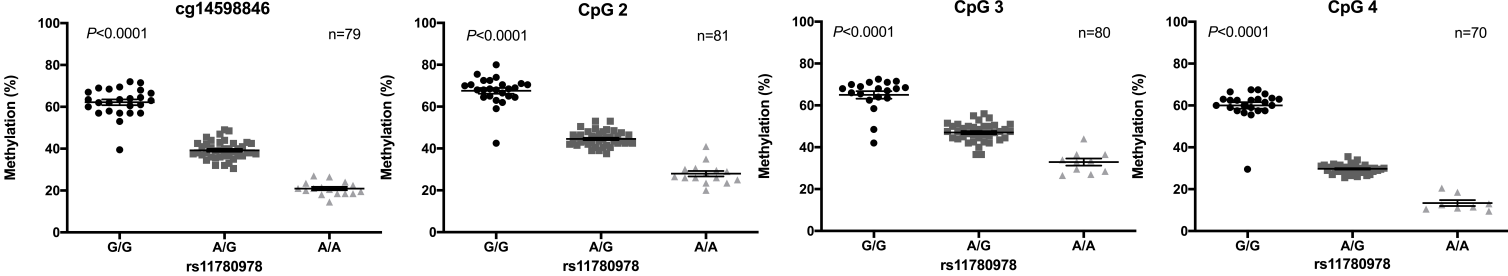

Supplement: Supplementary Fig. 4 — Association between rs11780978 genotype and methylation at CpG clusters cg19405177 (A) and cg14598846 (B) in synovium DNA. P values were calculated using the Kruskal–Wallis test. Horizontal lines and error bars show the mean ± SEM. n = the number of patients providing data per CpG site. [file mmc6.pdf]

Supplementary Figure 5

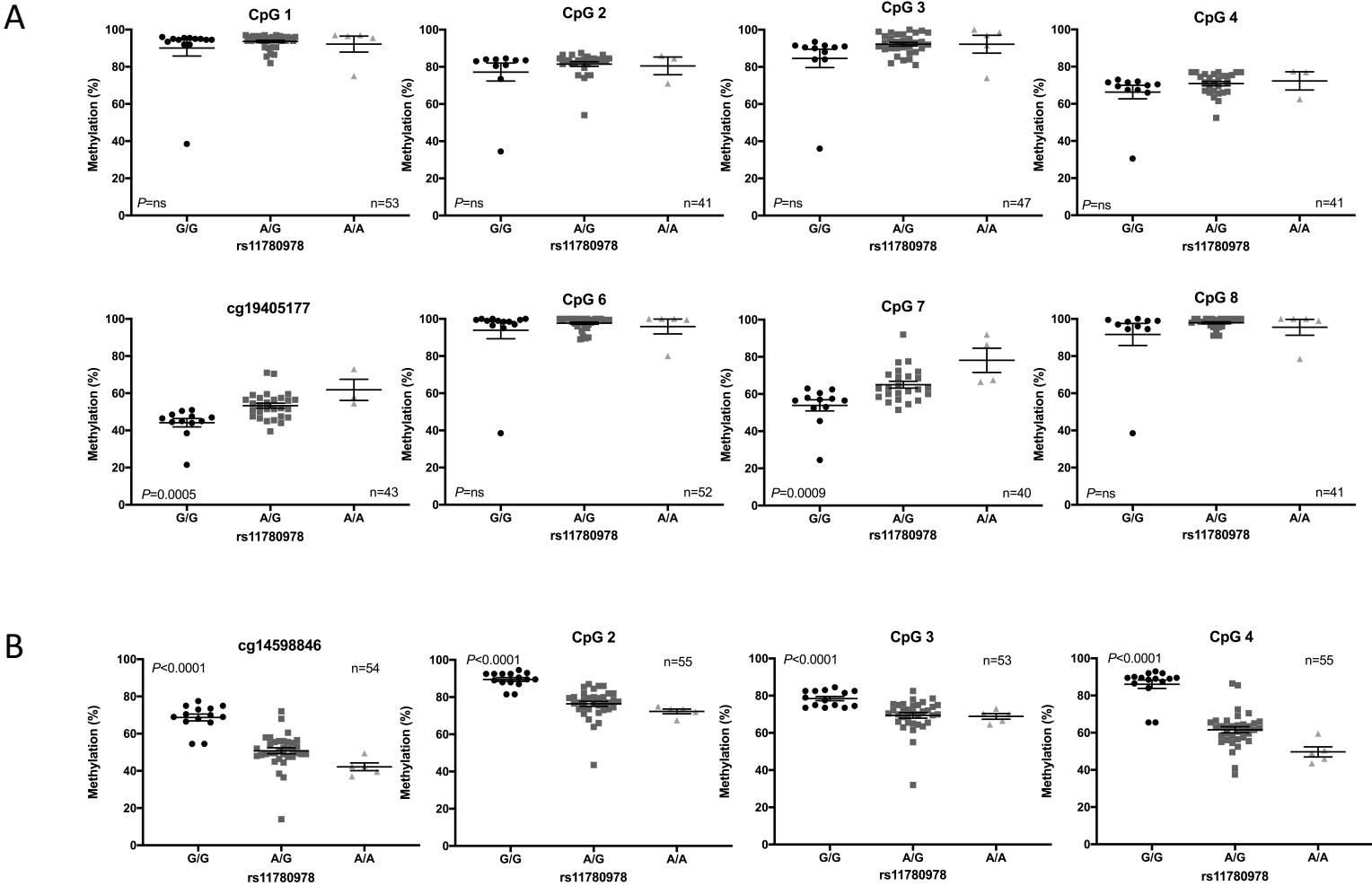

Supplement: Supplementary Fig. 5 — Association between rs11780978 genotype and methylation at CpG clusters cg19405177 (A) and cg14598846 (B) in blood DNA. P values were calculated using the Kruskal–Wallis test. Horizontal lines and error bars show the mean ± SEM. n = the number of patients providing data per CpG site. [file mmc7.pdf]

Supplementary Figure 6

A

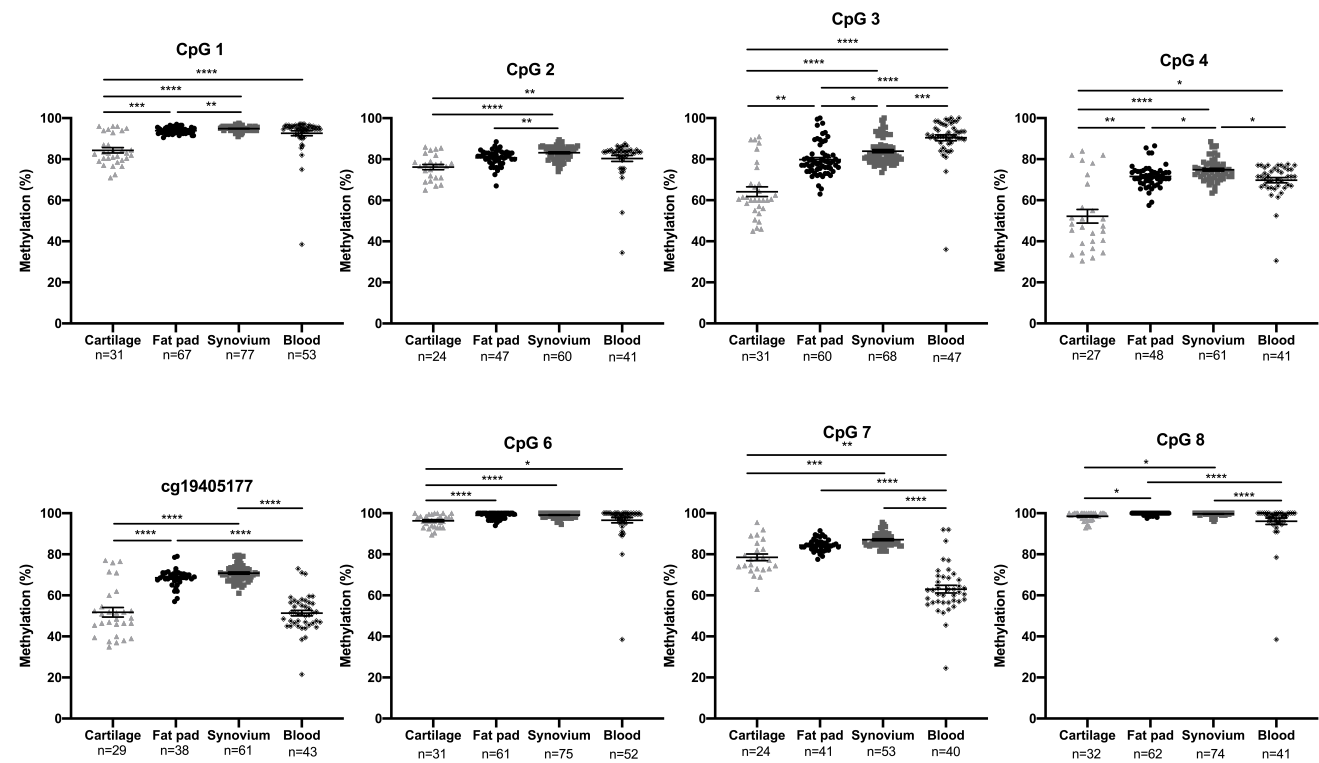

B

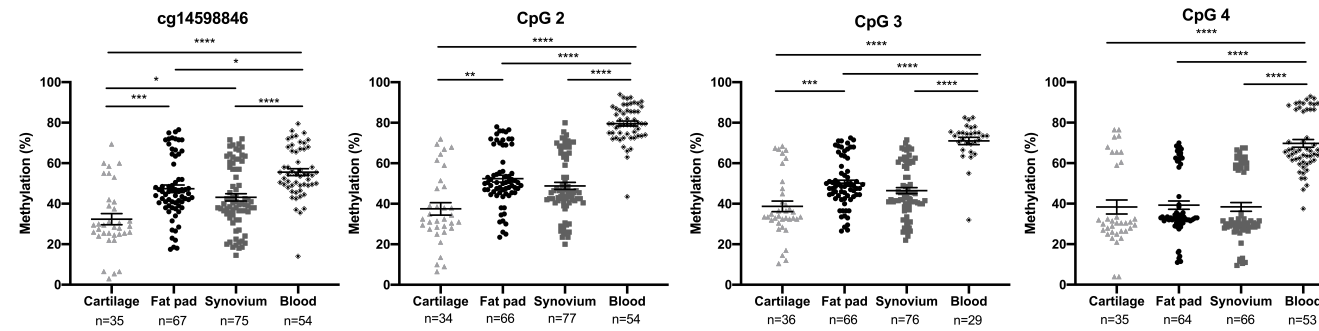

Supplement: Supplementary Fig. 6 — Methylation at CpG clusters cg19405177 (A) and cg14598846 (B) for the four patient tissues without stratification by rs11780978 genotype. P values were calculated using Kruskal–Wallis with Dunn's multiple comparisons. Horizontal lines and error bars show the mean ± SEM. n = the number of patients providing data per tissue and at each CpG site. ∗P ≤ 0.05; ∗∗P ≤ 0.01; ∗∗∗P ≤ 0.001 ∗∗∗∗P ≤ 0.0001. [file mmc8.pdf]

Supplementary Figure 7

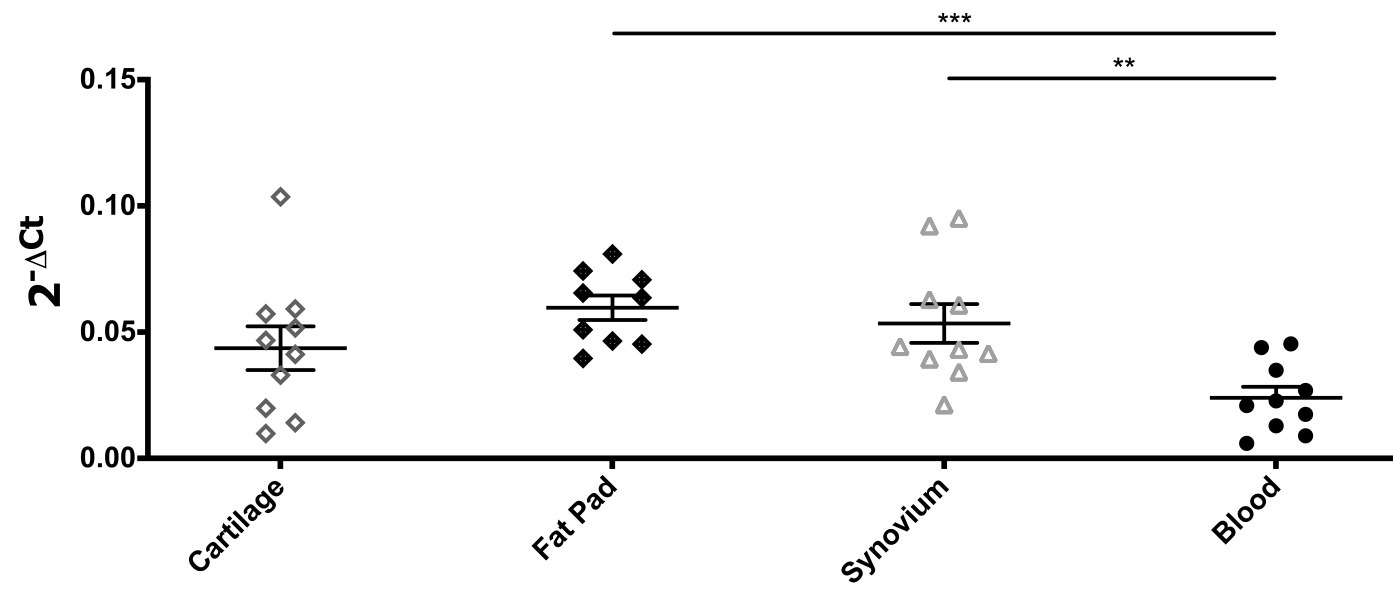

Supplement: Supplementary Fig. 7 — Expression of PLEC in tissue samples from OA patients. PLEC mRNA levels were measured by qPCR in cartilage (n = 10), fat pad (n = 9), synovium (n = 10) and blood (n = 10). Horizontal lines and error bars show the mean ± SEM. P values were calculated using a Mann–Whitney 2-tailed exact test. ∗∗P ≤ 0.01; ∗∗∗P ≤ 0.001 [file mmc9.pdf]

Supplementary Figure 8

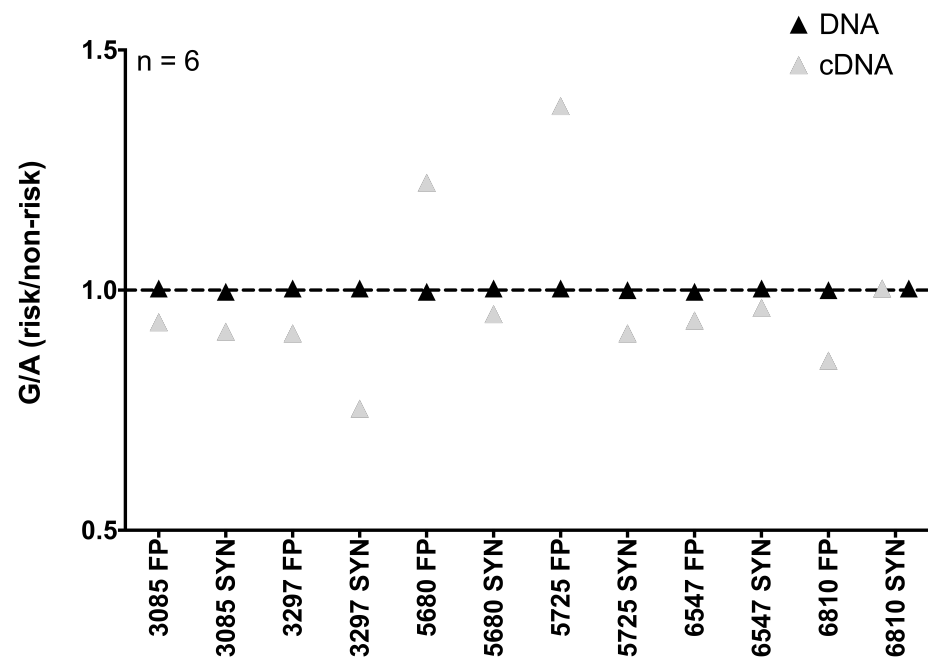

Supplement: Supplementary Fig. 8 — The six patients with AEI data for fat pad (FP) and synovium (SYN). The risk/non-risk allelic ratio is plotted, with a ratio <1 indicating decreased PLEC expression from the risk allele. For each patient, the mean of the DNA ratio (black, n = 3 technical repeats) and the mean of the cDNA ratio (grey, n = 3 technical repeats) is plotted. Numbers on the x-axis refer to the anonymised identification number assigned to patients at recruitment. [file mmc10.pdf]

Supplementary Figure 9

A

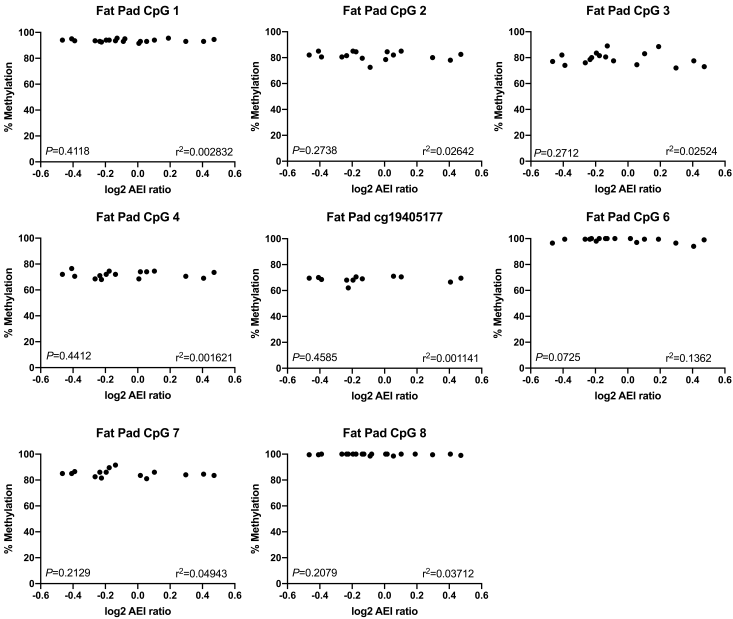

B

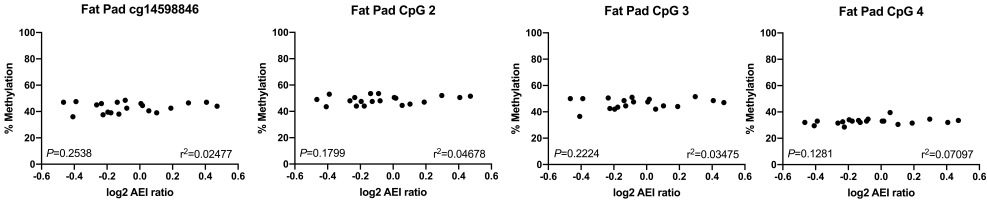

Supplement: Supplementary Fig. 9 — Methylation expression quantitative trait locus analyses in fat pad. PLEC log2 allelic expression imbalance (AEI) ratios were plotted against methylation at all CpGs in the cg19405177 (A) and cg14598846 (B) clusters. The square of the correlation coefficient (r2) and P values were calculated by linear regression analysis using a standard least squares model. [file mmc11.pdf]

Supplementary Figure 10

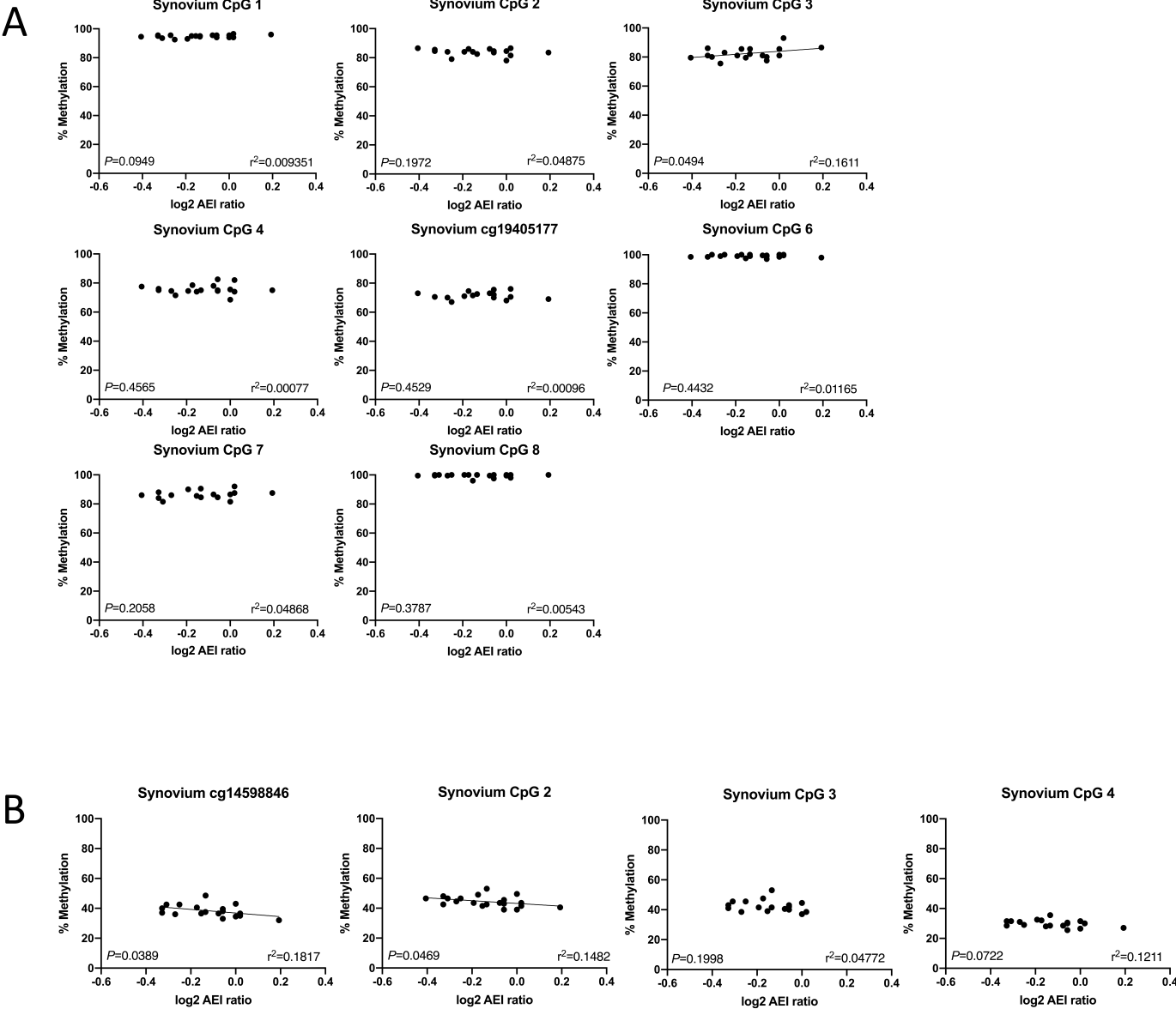

Supplement: Supplementary Fig. 10 — Methylation expression quantitative trait locus analyses in synovium. PLEC log2 allelic expression imbalance (AEI) ratios were plotted against methylation at all CpGs in the cg19405177 (A) and cg14598846 (B) clusters. The square of the correlation coefficient (r2) and P values were calculated by linear regression analysis using a standard least squares model. [file mmc12.pdf]
